# Ternary Mixture of Azoxystrobin, Boscalid and Pyraclostrobin Disrupts the Gut Microbiota and Metabolic Balance of Honeybees (*Apis cerana cerana*)

**DOI:** 10.3390/ijms24065354

**Published:** 2023-03-10

**Authors:** Jie Dong, Minjie Huang, Haikun Guo, Jiawen Zhang, Xiaodong Tan, Deqian Wang

**Affiliations:** 1 Institute of Animal Husbandry and Veterinary Science, Zhejiang Academy of Agricultural Sciences, Hangzhou 310021, China; 2 Institute of Agro-Product Safety and Nutrition, Zhejiang Academy of Agricultural Sciences, Hangzhou 310021, China

**Keywords:** azoxystrobin, boscalid, pyraclostrobin, metabolome, gut microbiota, *Apis cerana*

## Abstract

There is a growing risk of pollinators being exposed to multiple fungicides due to the widespread use of fungicides for plant protection. A safety assessment of honeybees exposed to multiple commonly used fungicides is urgently required. Therefore, the acute oral toxicity of the ternary mixed fungicide of ABP (azoxystrobin: boscalid: pyraclostrobin = 1:1:1, m/m/m) was tested on honeybees (*Apis cerana cerana*), and its sublethal effect on foragers’ guts was evaluated. The results showed that the acute oral median lethal concentration (LD_50_) of ABP for foragers was 12.6 μg a.i./bee. ABP caused disorder of the morphological structure of midgut tissue and affected the intestinal metabolism; the composition and structure of the intestinal microbial community was perturbed, which altered its function. Moreover, the transcripts of genes involved in detoxification and immunity were strongly upregulated with ABP treatment. The study implies that exposure to a fungicide mixture of ABP can cause a series of negative effects on the health of foragers. This work provides a comprehensive understanding of the comprehensive effects of common fungicides on non-target pollinators in the context of ecological risk assessment and the future use of fungicides in agriculture.

## 1. Introduction

The honeybee plays a vital role in global agriculture and the ecosystem balance, as it is the most important pollinator [1,2]. Over the past decade, the honeybee population has declined worldwide, which poses a major threat to agricultural production and ecological safety [3,4,5]. The large-scale use of agrochemicals such as herbicides, insecticides, fungicides, etc., is one of the most important factors causing honeybees to decline, as they cause irreversible damage to the populations of most honeybee species [6,7]. Therefore, there is a growing interest in assessing the risk of agrochemical exposure to honeybees.

Fungicides were introduced in the 1970s and have been widely used to control and prevent plant fungi in agriculture [8]. In recent years, accumulating evidence has detected the presence of multiple fungicides in many environments relevant to honeybee survival, such as flowers, pollen, honey, beebread, beeswax and honeybees themselves [9,10]. Among them, azoxystrobin, boscalid and pyraclostrobin most easily contaminate pollen and nectar [9]. Azoxystrobin and pyraclostrobin are common strobilurin-group fungicides that are widely used in agriculture to protect crops from fungal diseases. They mainly affect fungal mitochondrial respiration by blocking electron transport from cytochrome b to c1, which causes oxidative stress in the target fungus [11,12]. Both of these fungicides have a high level of bioactivity, a wide spectrum of activities and lower mammalian toxicity, and they occupy an important position in the agrochemical market [13,14]. Boscalid is an anilide fungicide, which inhibits fungal cell respiration (succinate dehydrogenase) by inhibiting mitochondrial respiration and blocking ATP production, thus controlling the growth of pathogenic fungi in a variety of crops [15]. It is often used in combination with the strobilurin-group fungicides (azoxystrobin and pyraclostrobin) and is most commonly applied to fruits and vegetables [16]. Although fungicides are not designed to target insects, the potential effects of non-insecticide fungicides on pollinators have received considerable attention.

Azoxystrobin, boscalid and pyraclostrobin are widely used fungicides in plants, which exert bacteriostatic effects by inhibiting the mitochondrial respiration of target microorganisms [17]. However, they could have certain effects on non-target organisms. Previous studies have shown that boscalid ingestion may affect the overall health of honeybees by reducing their forager ability [18]. Pyraclostrobin-contaminated beebread could induce cytotoxicity in the cells of the midgut [19]. Chronic exposure to pyraclostrobin not only altered the midgut morphology and reduced polysaccharide labeling in foragers [20], it also impaired the development, nutrient metabolism and immunity of larvae and pupae [21]. In addition, the majority of fungicides are provided as formulations, such as the commercial fungicide Pristine^®^, which consists of 25.2% boscalid and 12.8% pyraclostrobin and is produced by the BASF Corporation [22]. Several research studies on commercial fungicide have shown that a combination of boscalid and pyraclostrobin caused a negative impact on the nutrient absorption of workers, affecting their lifespan, foraging abilities, colony population size and olfactory learning abilities, as well as the disease resistance of the colony [23,24,25,26,27]. Moreover, recent evidence suggests that azoxystrobin, boscalid and pyraclostrobin fungicides and their binary mixtures significantly affect honeybees’ intestinal function [28]. Studies undertaken thus far provide single or binary fungicide evidence concerning the impact of honeybee physiology. However, what is not yet understood is the sublethal and synergistic effect of the ternary mixed fungicide of ABP (azoxystrobin, boscalid and pyraclostrobin) on honeybee gut microbes and metabolism.

*Apis cerana cerana* is the major native honeybee species in China and plays a vital role in ecological balance and agriculture industry. The aim of this study is to evaluate the effects of exposure to a ternary mixture of azoxystrobin, boscalid and pyraclostrobin (ABP) on the honeybee (*A. cerana cerana*). We determined the acute oral LD_50_ value of ABP for the first time and assessed the chronic toxicity of a sublethal dose (one-tenth dose of LD_50_) of the ABP fungicide to honeybees, including through midgut tissue morphology observation, midgut metabolome analysis, a gut microbial composition and function comparison, and immune- and detoxification-related gene expression detection. This research will enhance our understanding of the potential interactive effects of exposure to the combination of multiple fungicides on non-target organisms, contribute to a comprehensive assessment of the synergistic effects of multiple fungicides on ecological and environmental security, and provide a foundation for further exploring the molecular mechanisms underlying the effects of multiple fungicides on honeybee physiology.

## 2. Results

### 2.1. The Acute Oral LD_50_ of Ternary Mixed Fungicides in Apis cerana

**Figure 1:**
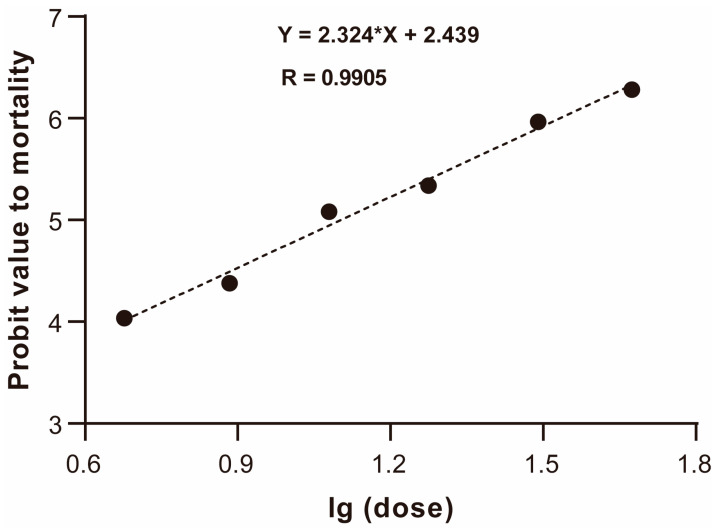
Toxicity regression curve of ternary mixed fungicide of ABP (azoxystrobin: boscalid: pyraclostrobin = 1:1:1, m/m/m) on *Apis cerana* by acute oral toxicity test.

To assess the acute toxicity of the ternary mixed fungicide of ABP (azoxystrobin: boscalid: pyraclostrobin = 1:1:1, m/m/m) on honeybees, and to determine the subsequent fungicide exposure concentrations, we performed a 48 h acute oral toxicity test. The results showed that the oral LD_50_ for 48 h of ABP was 12.6 μg a.i./bee (CL95% = 11.6–13.7, Figure 1), which is considered to be a low toxicity to *A. cerana* foragers. The dose of one-tenth of the LD_50_ was calculated and determined in the subsequent risk assessment experiments of ternary mixed fungicides in honeybees.

### 2.2. Fungicide Exposure Alters Midgut Morphology

**Figure 2:**
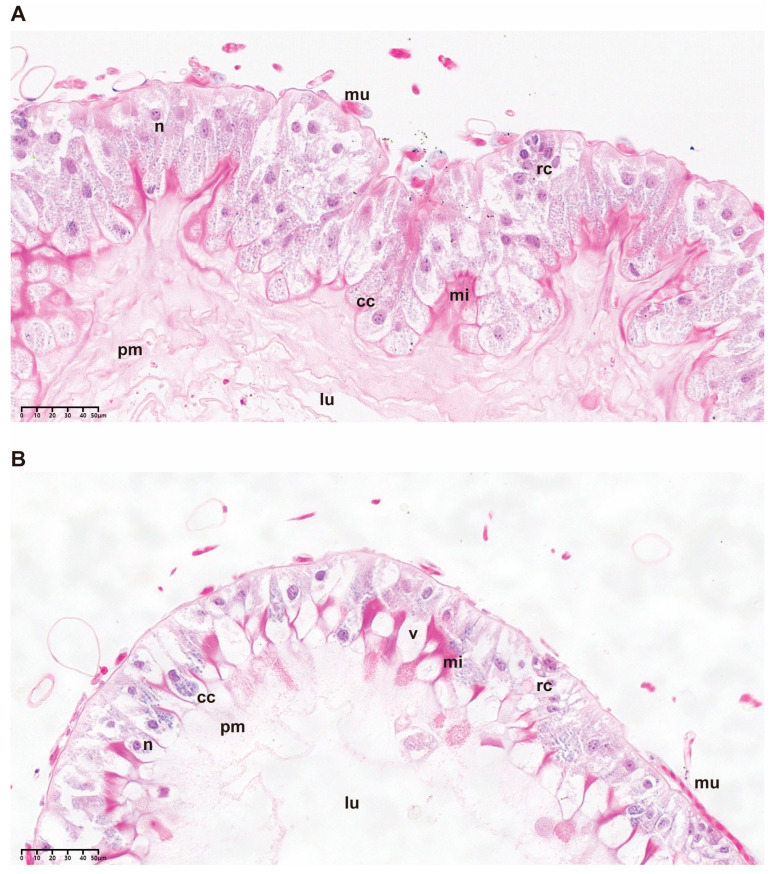
Light micrographs of the midgut of honeybees after 10 d of oral continuous exposure to ABP. (**A**) CTL—acetone syrup control; (**B**) ABP—0.063 mg/mL of fungicide mixture (azoxystrobin: pyraclostrobin: boscalid = 1:1:1, m/m/m). Legend: mu = muscle, n = nucleus, v = vacuole, cc = columnar cells, lu = lumen, mi = microvilli, pm = peritrophic membrane, rc = regenerative cells.

In the CTL group, the midgut epithelium of *A. cerana* was formed by a single layer of columnar cells (cc) with nuclei (n) rich in decondensed chromatin; it was shown to have well-defined microvilli (mi) and well-developed apical striated borders (Figure 2A). Nests of morphologically normal regenerative cells (rc) were scattered in the basal portion of the epithelium. In addition, honeybees in the CTL group showed multiple layers of peritrophic membrane (pm) secreted by the midgut cells into the intestinal lumen. Histological analyses of midguts of foragers exposed to ABP for 10 days presented histopathological damage, which was in clear contrast to the CTL group (Figure 2B). The epithelium was structurally disorganized with increased intercellular spaces, disorganized striated borders of microvilli formation and a reduced number of peritrophic membrane layers. Intense vacuolization was observed in digestive cells and nests of regenerating cells.

### 2.3. Fungicide Exposure Affects Amino Acid Metabolism

**Figure 3:**
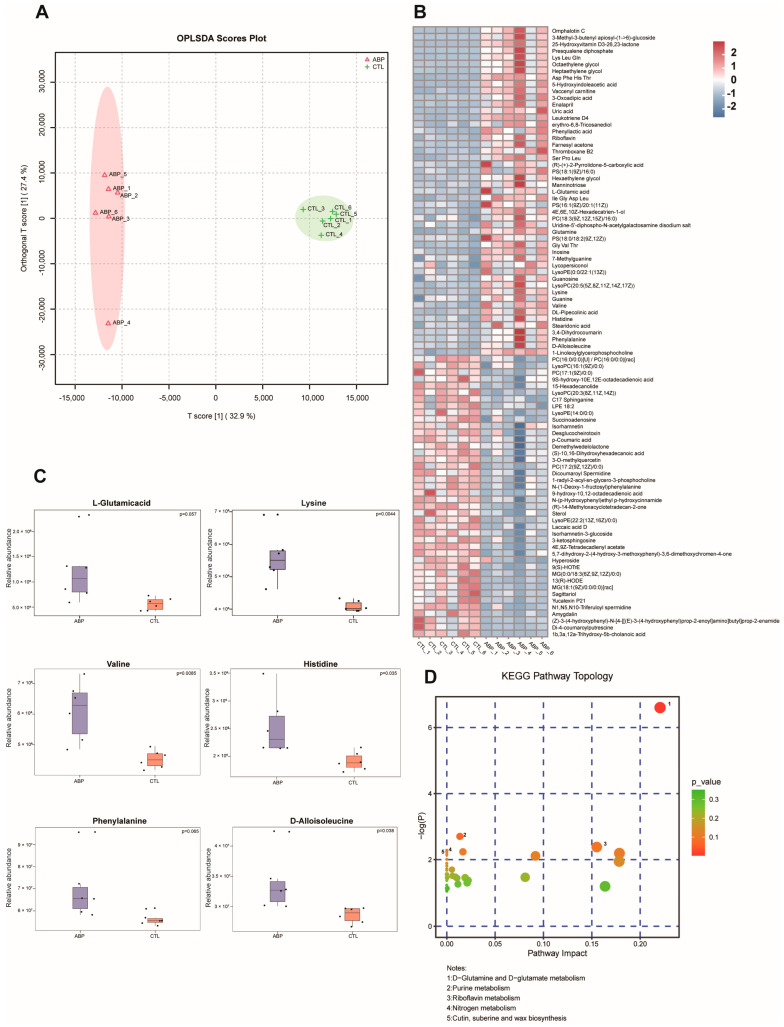
(**A**) Orthogonal partial least squares discriminant analysis (OPLS-DA) score plot between ABP-treated and control honeybees. (**B**) The heatmap visualization of the significant different metabolites between the ABP and CTL groups. Relative abundance is represented as color range from low level (−2, blue) to high level (2, red). (**C**) Fold change analysis describing alteration trend in computed means of related metabolites in ABP (purple bars) compared against the CTL (red bars). (**D**) Summary diagram of the pathway analysis with MetPA.

The OPLS-DA score plot of the ABP compared against the CTL group revealed a clear separation, which suggests the distinct metabolic properties of these two honeybee groups (Figure 3A). The evaluation of discriminatory metabolites from the ABP and CTL groups was then supplemented with the variable importance in the projection (VIP) and fold change (FC) analysis, with the aiming of comparing the differential metabolites that resulted from fungicide exposure. We identified 91 differential metabolites, of which 49 were upregulated and 42 were downregulated (Figure 3B). As shown in Figure 3C, six metabolites demonstrated trends in metabolite changes, which including L-glutamic acid, lysine, valine, histidine, phenylalanine and D-alloisoleucine. To explore the potential effects of ABP treatment on honeybees, KEGG pathway analyses of differential metabolites were carried out. The results demonstrated that D-glutamine and D-glutamate metabolism, as well as purine metabolism, were involved in the response to ABP treatment (Figure 3D).

### 2.4. Fungicide Exposure Disturbs Gut Microbial Balance

**Figure 4:**
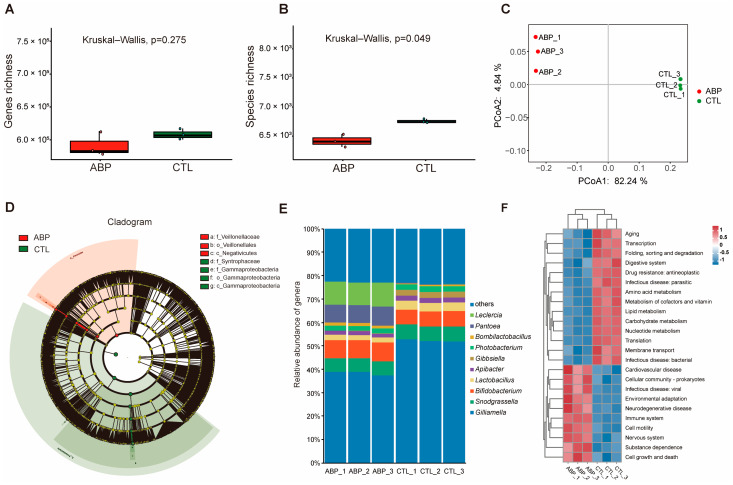
Effects of ternary mixed fungicide of ABP (azoxystrobin: boscalid: pyraclostrobin = 1:1:1, m/m/m) on gut microbial communities and function of *A. cerana cerana*. Comparison of the (**A**) gene richness and (**B**) species richness between fungicide exposure and CTL group. Each group includes three experimental repeats. (**C**) Principal coordinate analysis (PCoA) shows beta diversity among gut microbiota of fungicide exposure and CTL group based on Bray–Curtis distance. (**D**) Taxon differences in the gut microbiota between ABP and control group. Sizes of each dot is proportional to taxon abundance. Red-shaded areas indicate ABP-enriched taxa; green-shaded areas indicate the CTL-enriched taxa. Only the taxa that meet a significant LDA threshold value of >3 are shown. (**E**) Stacked bar plot of relative abundances of main bacteria at the genus level for each sample. (**F**) Hierarchical clustering heatmap of KEGG functional pathways from all samples. An increasing relative abundance is indicated by a color gradient from blue to red.

To further investigate the influence of the ternary mixed fungicide of ABP on honeybees, the composition and function of gut microbiota in foragers were assessed using metagenomic technology. The rarefaction curves of all the samples approached saturation in the rarefaction analysis, indicating that the large majority of *A. cerana* gut microbial genes were present in our gene catalogues, and the coverage was sufficient for subsequent community structure analyses (Figure S1). The results showed that, compared with control foragers, the gene richness and species richness of the gut microbiota in foragers exposed to the ABP fungicide exhibited a decreasing trend. Thus, the species richness in the intestinal flora of foragers in the ABP group was significantly lower than that of the normal foragers (CTL) (*p* < 0.05) (Figure 4A,B). Principal coordinate analysis (PCoA) based on Bray–Curtis distances revealed a clear separation of samples between the ABP exposure group and CTL group, demonstrating that the structure of the gut microbiota was obviously changed by fungicide exposure (Figure 4C). Fungicide exposure not only affected gene and species richness, but also the taxon relative abundance. Figure 4D shows a representative cladogram of the most differentially abundant taxa, demonstrating the changes in gut microbiota composition in the ABP exposure group. A total of seven taxa showed significant differences in their relative abundance among the ABP and CTL groups (LDA score > 3.0, *p* < 0.05). Based on the comparison of gut microbial community composition in each group at the genus level, five core bacterial genera, *Gilliamella*, *Bifidobacterium*, *Snodgrassella*, *Lactobacillus* and *Apibacter*, were dominate in all samples. However, the relative abundances of these five core genera obviously changed between the ABP and CTL groups, with the relative abundance of the *Gilliamella* and *Lactobacillus* genera showing a specially marked decrease in the ABP group. In addition, compared with the CTL group, the relative abundances of the *Leclercia* and *Pantoea* genera increased drastically, while the *Gibbsiella* genus decreased significantly in the ABP group (Figure 4E).

Usually, functional gene profiles tend to be altered when gut bacteria composition is perturbed. For this reason, the effect of fungicide exposure on the gut microbiota function was further observed by a KEGG pathway analysis on the enriched genes. The results show that there was a significant difference in the function of intestinal microbiota in honeybees between the ABP and CTL groups (Figure 4F). Compared with the CTL group, the functions associated with digestion and absorption (digestive system), as well as substance metabolism (such as carbohydrate metabolism, lipid metabolism and amino acid metabolism), of the gut microbiota in honeybees were significantly decreased, while the functions associated with immunity, environmental adaptation and diseases were significantly increased in the ABP group.

### 2.5. Fungicide Exposure Alters Gene Expression

**Figure 5:**
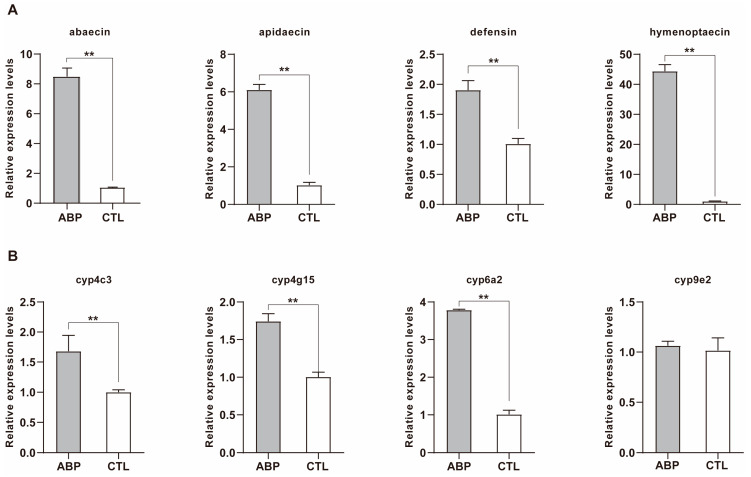
Relative expression of the (**A**) antimicrobial peptide family and (**B**) cytochrome P450-dependent monooxygenase genes of *A. cerana* foragers fed on ternary mixed fungicide (ABP) and 50% sucrose as control (CTL) for 10 days. Asterisks indicate differences between treatments via the *t*-test (** *p* < 0.01).

In order to understand the potential effect of ABP on *A. cerana*, certain important genes were selected for RT-PCR. As shown in Figure 5A, antimicrobial peptide family genes (*abaecin*, *apidaecin*, *defensin1* and *hymenoptaecin*) were significantly upregulated compared with those in the CTL group after exposure to ABP (*p* < 0.01). Transcripts of cytochrome P450-dependent monooxygenase in the ABP-treated group were all significantly upregulated (*p* < 0.01), whereas *cyp9 e2* showed no difference compared with the CTL group (Figure 5B).

## 3. Discussion

Honeybee pollination is critical to global agriculture and the ecosystem balance [1,2]. A wide range of fungicides are mainly used in agricultural practices to prevent and control fungal diseases in crops. At the same time, they pose a threat to honeybee health. In particular, the risks of fungicides that have a low toxicity and are non-lethal to bees are easily overlooked. Azoxystrobin, boscalid and pyraclostrobin, as effective ingredients in multiple formulas of agricultural fungicides, are widely used to control the diseases of fruits and vegetables [12,18,29]. However, accumulating evidence suggests that multiple mixed fungicides are present in flowers, nectar, pollen, beehives and even forager bees [9]. Therefore, it is highly necessary to evaluate the health risks associated with these fungicide mixtures for honeybees.

Our study is the first to identify the oral acute toxicity of the ternary mixed fungicide of ABP for *A. cerana*. Based on data from the IUPAC Pesticides Properties DataBase (PPDB), the oral acute LD_50_ values of azoxystrobin, boscalid and pyraclostrobin are >25 μg a.i./bee, >166 μg a.i./bee and >110 μg a.i./bee, respectively, indicating moderate toxicity (azoxystrobin) and low toxicity (boscalid and pyraclostrobin) for honeybees (*Apis mellifera*) [30]. Our study found that the oral LD_50_ of ABP for *A. cerana* foragers was 12.6 μg a.i./bee, which is lower than the LD_50_ values of the three fungicides individually for *A. mellifera*, indicating that the toxicity of the ternary mixed fungicides on eastern honeybees was greater than that of each single fungicide on western honeybees. This difference could be related to the species of honeybees. However, another study on the toxicity of these fungicides in eastern honeybees showed that the oral acute LD_50_ values of azoxystrobin, boscalid and pyraclostrobin were 12.7 μg a.i./bee, >119 μg a.i./bee and 36.6 μg a.i./bee, respectively, for *A. cerana* [28], which were higher than the LD_50_ value of ABP, suggesting that the toxicity of the multiple fungicide mixture was higher than each fungicide individually. These results are in agreement with those obtained regarding the toxicity of commercial fungicides (multi-fungicide mixtures) to earthworms, indicating that the sum of the toxicities of their active ingredients may be far higher than when assessed individually [31].

Although the fungicide has a low toxicity or may even be non-lethal to honeybees, its effects on important physiological functions, such as the digestion, absorption and metabolism of honeybees, cannot be ignored. Midgut morphological observation revealed that the azoxystrobin, boscalid and pyraclostrobin mixed fungicide caused visible changes in digestive cells in honeybees. This result was similar to that of honeybees exposed to a strobilurin fungicide, which induced digestive cells to present vacuolization and caused the nest of regenerative cells to have an abnormal morphology [20,32]. The abnormality or death of digestive cells may impair honeybee midgut digestion and nutrient absorption when chemicals accumulate [33]. Midgut enterocytes secrete into the intestinal lumen, forming the peritrophic membrane of a multilayered meshwork under normal conditions. In the present study, the layers of the peritrophic membrane decreased after exposure to the mixed fungicide for ten days. In addition to the physical barrier protecting the midgut from damage by food particles and pathogenic microorganisms, the peritrophic membrane can also improve absorption efficiency and participate in initial digestion to produce oligosaccharides and polypeptides [34,35]. Consequently, our results indicated dysfunction in the digestion and absorption of nutrients by honeybees exposed to the fungicide, even against food particles and pathogenic microorganisms.

In our study, the results of the OPLS-DA demonstrated a clear separation of metabolites between the ABP-treated and CTL groups, indicating metabolomic alteration induced by fungicide exposure. Six amino acids, including L-glutamic acid, lysine, valine, histidine, phenylalanine and D-alloisoleucine, were found to increase in the ABP-treated bees. Studies demonstrated the beneficial role of amino acids in colony growth, honeybee development and immune response [36,37,38]. Supplementation with phenylalanine, alloisoleucine, lysine and valine promoted honeybee gland and muscle development in cages and colonies [39]. However, the levels of these several amino acids were significantly higher in the fungicide group than in the control group. This might be because the ability of honeybees to utilize essential amino acids under fungicide exposure is diminished. Alterations in the amino acid metabolism were likewise observed in a previous study on the response of *Apis mellifera* L to thiacloprid [40]. Overall, the obtained metabolite data demonstrate that a sublethal dose of fungicide affected normal metabolism in honeybees.

As discussed above, the ABP fungicide clearly affected the intestinal tissue morphology and metabolism in foragers. Increasing numbers of studies have noted the importance of maintaining the integrity of the gut microbiota structure and function in honeybees for their health and well-being [41,42,43]. Therefore, the effects of the ternary mixed fungicide of ABP on the gut microbiota of honeybees were evaluated. This study found that there were seven taxa with significant differences in their relative abundance between the ABP and CTL groups. Among them, the abundance of taxa in Gammaproteobacteria and Syntrophaceae were significantly lower in the ABP group than in the CTL group. Gammaproteobacteria belongs to the Proteobacteria phylum, which is the most abundant phylum among the classified bacterial phyla in honeybee intestinal microbiota [44,45]. Fungicide exposure disturbed the main bacteria community composition in the honeybee gut. Therefore, fungicides might affect the function of intestinal microorganisms, and thus threaten bee health. Additionally, as the Veillonellaceae and Negativicutes taxa were significantly increased in the ABP group, their roles in honeybees still need further investigation.

Considering the differences at the levels of taxa between ABP and the CTL, the intestinal microbial composition of honeybees was further analyzed at the genus level. These results are in keeping with previous observational studies [46,47,48], which indicated that there are five core bacterial genera in the gut microbiota of *A. cerana*. However, the relative abundance of the core bacterial genera decreased due to the treatment with the ABP fungicide, such as that of *Gilliamella* and *Lactobacillus*, while that of the *Leclercia* and *Pantoea* genera was increased, making them the dominant genera in the gut microbiota of honeybees. Zheng et al. confirmed that the *Gilliamella* genus plays an important role in enhancing honeybees’ dietary sugar tolerance, which can help honeybees metabolize sugars they cannot otherwise metabolize, such as arabinose, mannose, rhamnose and xylose [49,50]. Moreover, previous studies have also shown that the *Lactobacillus* genus plays a significant role in processing complex carbohydrates, and that it prevents pathogens and maintains the health of honeybees [51,52,53]. One unanticipated result was that the *Leclercia* and *Pantoea* genera became the dominant bacterial genera in the ABP group. These two genera were found to be a kind of opportunistic pathogen that may cause septicemia and wound infection [54,55]. These findings were in accord with recent studies by Huang et al. [28], which implied that fungicide exposure may cause negative effects on honeybee health by reducing the abundance of beneficial bacteria and elevating the abundance of pernicious bacteria in the gut. This result might hint at a possibility of the development and utilization of probiotics to decrease the hazards of fungicides to honeybees. In our research, the analysis of the effect of the KEGG pathway on the gut microbiome further confirmed that ABP fungicide exposure had negative consequences for the physiological functions of intestinal microbiota, especially in reducing metabolism and immune defense functions. According to these data, we can infer that fungicide exposure disturbs the composition of the gut microbiome to alter the formal functions and poses a threat to honeybee health.

To further understand other potential effects of ABP on honeybee physiology, the transcript expression of the antimicrobial peptide family and cytochrome P450-dependent monooxygenases were examined. The antimicrobial peptide *abaecin* is a major antibacterial response peptide in the honeybee that is responsive to natural infections by the bacterial pathogen *Paenibacillus larvae* and parasite *Nosema ceranae* [56,57,58]. *Abaecin* is considered to be a backup for the potential emergence of bacterial resistance against the major immune component *apidaecin*. Additionally, *hymenoptaecin* complements *apidaecin* to inhibit the growth of certain apidaecin-resistant Gram-negative bacteria [59]. In contrast, *defensin*, which specifically targets Gram-positive bacteria [60], may play an important role in maintaining the immune competence of honeybees in the later stages of infection [61]. *A. cerana* possesses distinct abilities that can mount an innate immune response against neonicotinoid insecticides, and which shows a higher gene expression of these antimicrobial peptides [62]. Our results were consistent with that study, finding that four of the immune-related genes were all significantly upregulated after exposure to ABP. Different patterns of antimicrobial peptide expression implied distinct immune responses of *A. cerana* to ABP. Cytochrome P450-dependent monooxygenases are important detoxification enzymes that metabolize endogenous compounds and xenobiotics in insects [63]. In our study, ABP increased the expression of cyp4 c3, cyp4 g15 and cyp6 a2, which suggests that the expression of these genes may be related to the degradation of azoxystrobin, boscalid and pyraclostrobin. Although the targets of fungicides are not honeybees, these fungicides may also cause immune responses and detoxification in bees.

## 4. Materials and Methods

### 4.1. Chemicals and Honeybees

#### 4.1.1. Fungicide and Reagent

Azoxystrobin, boscalid and pyraclostrobin were purchased from Sigma-Aldrich (St. Louis, MO, USA). The ternary mixed fungicide of ABP is composed of azoxystrobin, boscalid and pyraclostrobin in an equal mass ratio (azoxystrobin: boscalid: pyraclostrobin =1:1:1, m/m/m). Stock solutions of the ternary mixed fungicide of ABP were prepared by dissolution in acetone and were stored at 4 °C for further dilution. Methanol, acetonitrile and acetic acid (LC-MS grade) were purchased from ANPEL (Shanghai, China). The PrimeScript™ RT reagent Kit with the gDNA Eraser and TB Green^®^ Premix Ex Taq™ II were obtained from TaKaRa (Dalian, China).

#### 4.1.2. Honeybee Preparation

Five queen-right and healthy colonies of *Apis cerana* from the apiary at the Zhejiang Academy of Agricultural Sciences (Hangzhou, China) were used in the experiment. The chosen colonies were not exposed to pesticides or agricultural activities. In this study, the forager bees were used to perform fungicide risk assessments, as their foraging behavior was the primary route for exposing the colony to the environment. Newly emerged worker bees were collected from a normal colony and marked with paint, and then were returned to their colonies. After 20 days, we collected the marked 20-day-old forager bees that carried pollen to the entrances of hives during the morning, as described by Capela et al. [64]. Then, all the foragers were placed randomly into cages (11 cm × 11 cm × 11 cm, n = 60 bees per cage) with a feeder. They were fed 50% (*w*/*v*) sucrose solution ad libitum for at least 2 h to synchronize their diet state. All bees were maintained in a climate-controlled incubator (33 ± 1 °C, relative humidity (RH) 60 ± 10%) under a dark condition until used.

### 4.2. Acute Oral Toxicity Test (LD_50_)

Determination of the acute oral LD_50_ was carried out for *A. cerana* based on the OECD Guidelines, #213 [65]. Experiments were performed in a dark room with a temperature and relative humidity of 25 ± 1 °C and 60 ± 10%, respectively. Firstly, the stock solution of the ABP fungicide was diluted into six exposure doses, from 2.540 to 0.243 g a.i./L, by means of successive dilutions in a geometric series, using the 50% (*w*/*v*) sucrose solution (containing 5% acetone and 0.1% Tween 80). Secondly, the collected 20-day-old foragers were randomly assigned to 21 test cages in seven groups (11 cm × 11 cm × 11 cm, n = 10 bees per cage) and starved for 2 h before the initiation of the test. Then, 200 μL of the 50% (*w*/*v*) sucrose solution containing the test fungicide was provided to each group. Each exposure dose was set up in three replicate cages (one cage with 10 bees). At the same time, the control group (CTL) was also set up in three replicate cages and provided with the 50% sucrose solution containing no more than 5% acetone and 0.1% Tween 80 in the final volume. At last, the amount of this diet consumed per group was measured. After 6 h, the 50% (*w*/*v*) sucrose solution containing the test fungicide for each group was replaced by the 50% sucrose solution alone. During the 48 h test, toxicity signs and mortality were observed and recoded every 24 h.

### 4.3. Fungicide Exposure

One-tenth of the LD_50_ concentration of ABP, obtained with the acute oral toxicity test, was selected for the fungicide exposure experiment. The collected 20-day-old foragers were randomly divided into a ternary mixed fungicide exposure group (ABP) and a control group (CTL). There were six replicates (six test cages per group, n = 30 bees per cage) for each group. The stock solution of ABP was diluted to 0.063 mg/mL with the 50% (*w*/*v*) sucrose solution for use in the fungicide exposure experiment. Solvent promoter Tween 80 was added to a final concentration of 0.1%, and the final concentration of acetone did not exceed 5%. Before the beginning of the exposure assay, the foragers were starved for 8 h. After that, the ABP and CTL groups were given the ABP sucrose solution and 50% sucrose solution (*w*/*v*) (containing 5% acetone and 0.1% Tween 80), respectively. Each cage was fed fresh 1 mL test solutions every day. Each group was supplemented with the 50% sucrose solution (*w*/*v*) for feeding ad libitum when the test solution was completely consumed. All of the foragers were sampled on the tenth day.

### 4.4. Morphological Observation of the Midguts

After 10 d of fungicide exposure, the midguts of each experimental group were collected following CO_2_ anesthesia, dissected at room temperature and fixed in 4% paraformaldehyde for 24 h at 4 °C. Then, histological sections were dehydrated with ethanol, embedded in paraffin and stained with hematoxylin and eosin (HE) for morphological analysis.

### 4.5. Metabolite Extraction and Data Analysis

The 10 pooled honeybee midguts of each group were finely ground with a mortar and pestle in liquid nitrogen. The samples with 100 μL of ice-cold H_2_ O and five ceramic beads were homogenized using the homogenizer for 60 s. Then, 400 μL of methanol/acetonitrile (1:1, *v*/*v*) was added to the homogenized solution for metabolite extraction. The solutions were sonicated for 30 min at 4 °C. After centrifugation (20 min, 12,000× *g*, 4 °C), the supernatant was transferred to fresh microtubes and then freeze-dried. The freeze-dried samples were dissolved in 200 μL of 30% acetonitrile and transferred to insert-equipped vials for UHPLC-ESI-MS/MS analysis (UPLC, Vanquish; MS, QE). To monitor the stability and repeatability of the instrument analysis, quality control (QC) samples were prepared by pooling 10 μL of each sample and analyzing them together with the other samples.

The raw MS data were acquired with the Q Exactive using Xcalibur 4.1 (Thermo Scientific, Waltham, MA, USA), and processed using Progenesis QI (Waters Corporation, Milford, CT, USA). Quantified data were output into excel format. Data were analyzed with the R package, where they were subjected to multivariate data analyses, including principal component analysis (PCA) and orthogonal partial least squares discriminant analysis (OPLS-DA). The 7-fold cross-validation and response permutation testing was used to evaluate the robustness of the model. The variable importance in the projection (VIP) value of each variable in the OPLS-DA model was calculated to indicate its contribution to the classification. Metabolites with a VIP value >1 and fold change >1.5 or <2/3 were further applied to Student’s *t*-test at a univariate level to measure the significance of each metabolite; *p*-values less than 0.05 were considered to be statistically significant. The pathway analysis of annotated metabolites was performed with IPA (http://metpa.metabolomics.cn/Metpa/faces/Home.jsp (accessed on 5 June 2022)) based on the Kyoto Encyclopedia of Genes and Genomes (KEGG) database (http://www.kegg.jp/kegg/pathway.html (accessed on 5 June 2022)).

### 4.6. Gut Microbiome Analysis

#### 4.6.1. Sample Collection

The hindgut contents of forager bees were collected according to the method reported by Ellegaard et al. [47]. First, the gut of each collected bee was carefully drawn out of the abdomen. After removing the midgut and Malpighian tubules, the hindgut (ileum and rectum) was collected in bead-beating tubes with PBS (kept on ice). Second, hindgut tissue was homogenized with a bead beater using ceramic beads (15 s at speed 3.0). Then, by using a series of differential centrifugation, the bacterial cells in the hindgut contents were enriched while pollen and tissue debris were removed. Briefly, 1 mL of PBS was added to tissue homogenates of the hindgut; this was vortexed and mixed, then centrifuged at 500× *g* for 5 min at 4 °C. The supernatant was collected and pelleted by centrifugation at 10,000× *g* for 10 min at 4 °C. The precipitated microbe pellet was resuspended in PBS. The above operation of washing and centrifugation was repeated twice. The final precipitate of the bacterial pellet was collected and frozen at −80 °C until use.

#### 4.6.2. Metagenome Sequencing and Data Analysis

Each sample’s genomic DNA was isolated from 10 pooled hindgut contents following a CTAB-based protocol [66]. Triplicate samples were set up for each treatment. A total amount of 1 μg genomic DNA per sample was used as input material for the DNA library preparations. Based on the manufacturer’s recommendations, sequencing libraries were created using the NEB Next Ultra^TM^ RNA Library Prep Kit for Illumina^®^ (NEB, Ipswich, MA, USA), and index codes were added to attribute sequences to each sample. Then, six libraries were sequenced on the Illumina NovaSeq platform using the PE150 protocol by KaiTai Bio-Co., Ltd. (Hangzhou, China). All raw data are available through the Sequence Read Archive (SRA) database with accession number PRJNA837694 (https://dataview.ncbi.nlm.nih.gov/object/PRJNA837694 (accessed on 5 June 2022)).

Data processing and analysis were carried out as follows: High-quality clean reads were obtained after trimming adapter sequences, removing low-quality reads (Q score < 20) and discarding invalid reads containing poly-N (>10 bp). Then, clean reads were de novo assembled using MEGAHIT (v1.2.6) to obtain scaftigs [67]. Scaftigs (>500 bp) of each sample were analyzed by MetaGeneMark (v2.10) to predict open reading frames (ORFs) [68]. An initial non-redundant gene catalogue (nrGC) of each sample was obtained by using CD-HIT software (v4.5.8) and was based on the prediction of ORF results [69]. The reads from each sample were realigned to the nrGC using Bowtie (http://bowtie-bio.sourceforge.net/bowtie2/index.shtml (accessed on 8 September 2022)). The relative abundance was calculated using the read numbers of each non-redundant gene mapped to the initial nrGC. Principal coordinate analysis (PCoA) based on the Bray–Curtis distance was conducted using the APE library [70]. The taxonomic information of unigenes was obtained by using DIAMOND (v0.9) based on the NCBI microNR database (including bacteria, fungi, archaea and viruses, version: 12 June 2022) with blastp (e-value ≤ 1 × 10^−5^) [71]. Based on the lowest common ancestor-based algorithm (LCA) implemented in MEGAN, the taxonomic level of each gene was determined [72]. A Kruskal–Wallis test was used to compare the differences in genes and species richness in gut microbiota. LDA effect size (LEfSe) analyses were performed using the LEfSe tool (http://huttenhower.sph.harvard.edu/lefse/ (accessed on 15 September 2022)) to identify the greatest differences in taxon abundance between each group of samples. KEGG (Kyoto Encyclopedia of Genes and Genomes) pathway analysis of each gene was performed by using DIAMOND to compare against the KEGG database (http://www.kegg.jp/kegg/pathway.html (accessed on 20 September 2022)).

### 4.7. RNA Extraction, RT-qPCR and Statistics

Total RNA was isolated from each sample of 10 pooled honeybees (remaining body without gut) using the TRIzol reagent and was checked for purity and integrity. First-strand cDNA was obtained with the PrimeScript™ RT reagent Kit with the gDNA Eraser. Quantitative real-time PCR was performed on an ABI 7500 Real-Time PCR system (Applied Biosystem, Carlsbad, CA, USA) with the specific primers (Table S1) and the following parameters: 95 °C for 5 min, 40 cycles of 95 °C for 10 s, 60 °C for 34 s, 60 °C for 40 s and 60 °C for 5 min. Relative gene expression levels were quantified based on β-actin expression levels by using the 2^−ΔΔCt^ method with three independent biological replicates [73]. Data are presented as the mean ± standard deviation (SD). Statistical analysis was conducted using the one-way ANOVA or independent-samples *t*-test with the SPSS 22.0 software version 22.0 (IBM, Armonk, NY, USA), and data with *p*-values < 0.05 were considered statistically significant.

## 5. Conclusions

In this study, we evaluated the sublethal effect of exposure to a ternary mixture of azoxystrobin, boscalid and pyraclostrobin on honeybee health based on metabolome profiling and metagenomic data. We found that the toxicity of multiple fungicides mixed together as ABP was higher than that of each single component. Exposure to mixed fungicides causes serious damage to the midgut tissue structure, and affects the normal metabolism, especially amino acid metabolism. Furthermore, the balance of gut microbiota in honeybees was also disturbed by fungicides. The abundance of beneficial bacteria was reduced, and the abundance of harmful bacteria was increased, thus affecting the functions of metabolism and immune defense. The detoxification and immunity of the whole bodies of honeybees were at a high level. Overall, we demonstrated that exposure to a mixture of multiple fungicides could cause various negative impacts on honeybee health, which requires our attention. These results contribute to a better understanding of the potential hazards to honeybee health caused by the combination of multiple fungicides. It is helpful for people to consider the rational application of fungicides and provide new insights into how probiotics can be utilized to reduce the hazards of fungicides to honeybees.

## Data Availability

The data presented in this study are available upon request from the corresponding author.

## Supplementary Materials

The following supporting information can be downloaded at: https://www.mdpi.com/article/10.3390/ijms24065354/s1.
