# Supplementary material for: Ternary Mixture of Azoxystrobin, Boscalid and Pyraclostrobin Disrupts the Gut Microbiota and Metabolic Balance of Honeybees (Apis cerana cerana)
Source: Int J Mol Sci. 2023 Mar 10;24(6):5354. doi: 10.3390/ijms24065354 (PMC10049333; doi:10.3390/ijms24065354)
Supplement: Supplementary file 1 [file ijms-24-05354-s001.zip › Figure S1.pdf]

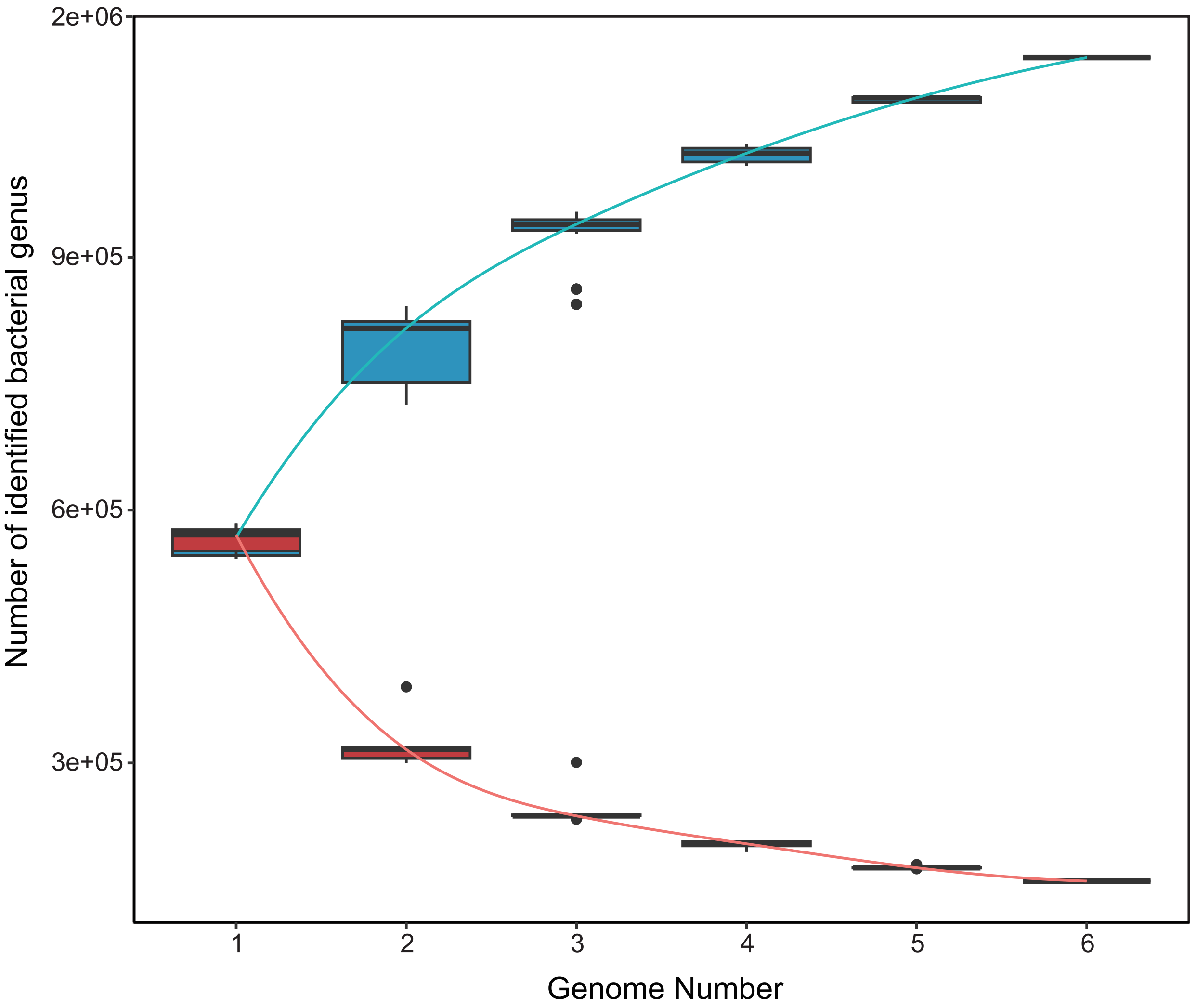

Figure S1: The rarefaction analysis of identified bacteria at genus level. Blue line represents the pan-genome, red line represents the core genome. Genome number represents 6 metagenome samples.
